# Understanding nonproliferative diabetic retinopathy progression using noninvasive imaging

**DOI:** 10.1038/s41433-025-03901-3

**Published:** 2025-07-11

**Authors:** José Cunha-Vaz, Luís Mendes, Débora Reste-Ferreira

**Affiliations:** 1 AIBILI - Association for Innovation and Biomedical Research on Light and Image, Coimbra, Portugal; 2 Faculty of Medicine, University of Coimbra, Coimbra, Portugal

**Keywords:** Prognostic markers, Retinal diseases

## Abstract

It is well accepted that only a subset of individuals with diabetes is expected to progress to advanced retinopathy and is at risk of losing functional vision. It is, therefore, of major relevance to identify this subset of patients and when they enter into rapid progression. The Early Treatment Diabetic Retinopathy Study (ETDRS) severity scale is the classic gold standard for grading diabetic retinopathy progression. The fundus abnormalities seen in Diabetic Retinopathy can conceptually be split into three main phenotypes. Those resulting from retinal neurodegeneration, those related to an alteration of the Blood-Retinal Barrier and, finally, those resulting from ischemia. In eyes showing the ischemic phenotype, disease progression is characterized by an initial stage of increasing hypoperfusion involving initially the superficial capillary plexus with progressive involvement of the deep capillary plexus followed by an hyperperfusion response consisting of dilated shunt vessels and intraretinal microvascular abnormalities. Visual acuity is generally maintained as the retinopathy progresses to loss of visual acuity as a result of either clinically significant macular oedema (CSMO) or proliferative diabetic retinopathy (PDR). It is the microvascular changes that occur in response to the progressive capillary closure and the hyperperfusion response characterized by abnormally dilated shunt vessels that create the conditions for CSMO and PDR. Our present understanding of the progress of diabetic retinal disease indicates that prevention of the major vision-threatening complications, may be addressed by either halting the progressive ischemia which characterises the initial hypoperfusion stage or by targeting the angiogenic and inflammatory response that follows.

## Introduction

Diabetic retinopathy (DR) is a frequent complication of diabetes and, through its vision-threatening complications, i.e., clinically significant macular oedema (CSMO) and proliferative diabetic retinopathy (PDR), may lead to blindness. Diabetes is regarded as a global epidemic. It is estimated that by 2045 there will be 783 million people worldwide affected by diabetes. Considering that a third of people with diabetes have signs of DR and that ~10% develop vision-threatening retinopathy and vision loss, it is one of the leading causes of blindness in working-age people [1].

However, the progression of nonproliferative diabetic retinopathy (NPDR) to vision-threatening stages, CSMO and PDR, varies from individual to individual. The cumulative occurrence of progression from mild NPDR to vision-threatening complications has been determined to be in the order of 14–16%. However, when moderate to severe retinopathy is already present, the progression to vision loss is in the order of 58% [2, 3]. It is, therefore, crucial to identify progression of retinopathy in a given patient and predict which patients with diabetes are at a high risk for progression to vision loss.

The gold standard method for staging DR progression is that of the Early Treatment Diabetic Retinopathy Study (ETDRS) grading. This method is based on the identification of a series of lesions mostly associated with microvascular disease [4]. This grading is based on solid long-term accumulated data. The ETDRS severity scale is not linear regarding the risk of developing complications such as vision loss. Eyes classified as moderate NPDR (ETDRS levels 43–47) have an 8.6% risk of progressing to PDR, while eyes classified as severe NPDR (ETDRS level 53) have a 45% risk of developing PDR. It is therefore of major relevance to characterize the progression of NPDR and to identify risk markers associated with disease progression [5]. In order to understand the progression of DR, it is necessary to examine and characterize the alterations occurring in the different ETDRS severity levels to identify the lesions that are predominant in the different retinopathy stages as the retinopathy progresses to vision-threatening complications.

## Main pathways of disease progression. Different Phenotypes of DR Progression

NPDR is characterized by the presence of microaneurysms, small haemorrhages, indirect signs of vascular hyperpermeability (hard exudates) and capillary closure (soft exudates or cotton-wool spots). These alterations are predominant in the fundus images during the first four initial stages of the retinopathy (ETDRS levels 10–43), according to the ETDRS classification.

It is recognized that the duration of diabetes and the level of metabolic control determine the progression of DR. However, these risk factors do not explain the great variability that characterizes the progression of retinopathy in different individuals. There are many diabetic patients who, after many years with diabetes, never develop vision-threatening retinal changes, while other patients progress rapidly [4, 6].

Clinically, DR is said to be present when microaneurysms and small haemorrhages appear on ophthalmoscopic examination. On histopathological examination, the vascular changes are initiated in the small vessels in the form of endothelial proliferation and pericyte and endothelial damage [7]. These initial lesions are focal and appear to be located mainly in the posterior pole of the retina. As the disease progresses, in a relatively small number of patients the capillaries of the arterial side of the retinal circulation show increased closure with cell loss and the areas of capillary closure enlarge [7, 8]. As they enlarge, they are seen to be crossed by remaining abnormally dilated capillaries, which appear to act as arteriovenous shunts, receiving the blood directed from the surrounding closed capillary net and the number of microaneurysms increase [9].

What remains to be fully understood are the mechanisms involved in triggering these lesions and the identification of the different stages of retinal vascular disease progression as retinopathy progresses and leads to vision loss.

Hyperglycaemia appears to be sufficient to initiate the development of DR as revealed by the development of retinopathy in animals experimentally made hyperglycaemic [10–12]. Consistently, a number of experimental studies have shown that intensive therapy sufficient to minimize hyperglycaemia inhibits the development of retinopathy [13]. However, the observation that not all patients with poor metabolic control develop advanced stages of retinopathy suggests that other factors, such as genetic predisposition, may determine individual susceptibility to the disease [14, 15].

As a first insight to the complexity of diabetic retinal disease, it is important to keep in mind that diabetes is not a single disease, but rather a group of conditions broadly categorized by a single diagnostic criterion, hyperglycaemia, on which disparate metabolic derangements converge [16]. Increasingly, there is evidence suggesting that type 2 diabetes (T2D), the predominant diabetes subtype making up 90-95% of cases, is itself heterogeneous in terms of both mechanisms of action and relationships with health outcomes. Clustering approaches using clinical or genetic biomarkers have identified subtypes of T2D that are clinically distinct and differentially associated with diabetic complications [17, 18].

Hyperglycaemia and genetic predisposition are associated with a variety of pathophysiological events identified in the progression of diabetic retinopathy [19]. To date, several major mechanisms are thought to induce retinal disease in DR, including 1) the polyol pathway, 2) non-enzymatic glycation, 3) activation of protein kinase C (PKC), 4) genetic factors, 5) inflammation and oxidative stress, all of which have been implicated in the development of microvascular damage and retinopathy. Because the retinal vasculature lacks autonomic innervation, modulation of blood flow is done through the surrounding neuronal and glial cells which makes it extremely susceptible to neuroglial disturbances [20].

The earliest alterations that can be detected by presently available methods of examination of the retina in diabetes are breakdown of the blood-retinal barrier (BRB), alterations in the neuroglial structure and function, and signs of capillary closure. These alterations can be detected before they are visible on ophthalmoscopy and characterize the preclinical stage of diabetic retinal disease [21–23]. These early changes occur with a background of neuroglial degeneration. Thinning of the ganglion cell layer plus inner plexiform layer (GCL + IPL) is observed in a large proportion of patients with diabetes and can be identified in the absence of alterations of the Blood-Retinal Barrier or evidence of capillary closure [24]. It outstrips the neurodegeneration associated with normal aging [25] and it appears to be primarily related to diabetes mellitus (DM) duration and not to HbA1C levels. Also, the breakdown of the blood-retinal barrier has been shown to be an early finding, even in the absence of clinically visible retinopathy. It was demonstrated by vitreous fluorometry in 1975 by our group [21] and this observation has now been confirmed using a more sensitive methodological approach [26].

Finally, microvascular closure and hypoperfusion demonstrated using fluorescein angiography or optical coherence tomography angiography (OCTA) are the predominant pathology in approximately another 25% of the eyes in the early stages of diabetic retinal disease [24].

These and other observations support the concept that dysfunction of the neurons and glial cells may induce changes in the neurovascular unit [27]. It is also of major relevance that retinal neurodegeneration appears to participate in the development of the early microvascular changes that occur in DR, such as breakdown of the BRB [28, 29], vascular regression [30] and impairment of neurovascular coupling [31]. A specific role for pericyte damage, resulting in loss of regulation of retinal vascular tone, has also been proposed [32, 33]. Finally, a large body of evidence supports the role of inflammation in the pathogenesis of the microvascular damage [34–36].

**Fig. 1:**
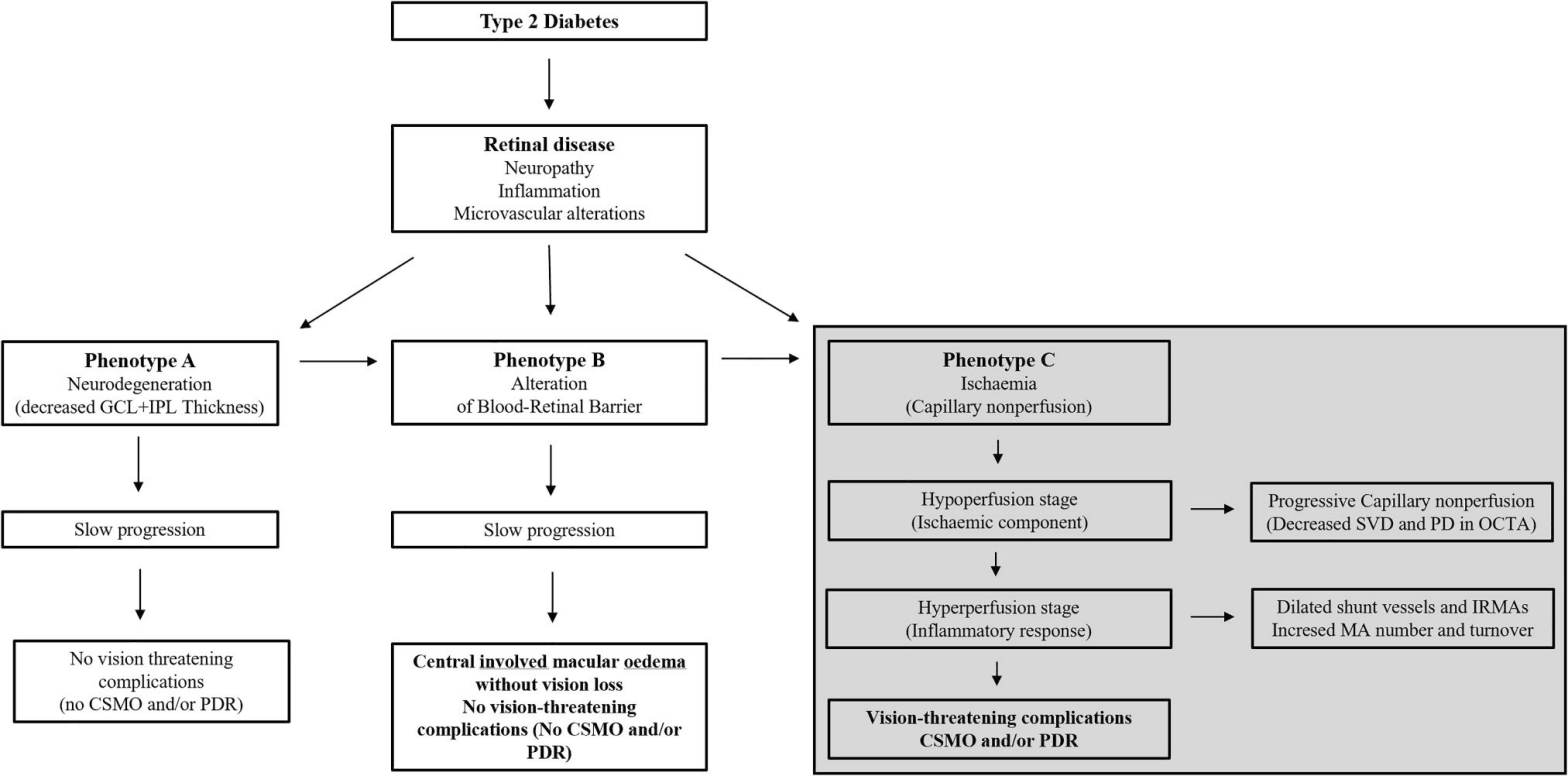
Pathophysiology of diabetic retinopathy: different pathways of disease progression.

In summary, the fundus abnormalities that characterize diabetic retinal disease can conceptually be split into three major categories: 1. those findings resulting from neuronal and glial degeneration that lead to vision alterations and condition the microvascular response; 2. those findings resulting from breakdown of the blood-retinal barrier, i. e., haemorrhages, lipid exudates and retinal oedema, 3. those findings resulting from ischaemia with subsequent overproduction of vascular growth factors, i.e. capillary closure, cotton-wool patches and intraretinal microvascular abnormalities (IRMA) (Fig. 1).

There are therefore three major pathways of disease in the diabetic retina that may occur as the initial stages of diabetic retinal disease: namely, neurodegeneration, breakdown of the blood-retinal barrier and microvascular closure. Each one of these pathways of disease may be dominant in different patients, conditioning the progression of the retinal changes and determining the clinical evolution of diabetic retinopathy in each patient [37]. In some eyes neurodegeneration, identified by thinning of the GCL + IPL, or multifocal electroretinogram mfERG changes, is the dominant alteration. In another set of patients the breakdown of the blood-retinal barrier dominates the clinical picture and is identified by subclinical macular oedema registered in OCT and finally, in another group of patients, capillary closure is present very early in the disease process, and dominates the clinical picture, identifying a ischaemic phenotype characterized by rapidly progressing capillary closure [38]. It remains unclear which diabetic metabolic alterations are responsible for each disease pathway and resulting phenotype.

There is also evidence suggesting that all these three major disease pathways for progression of retinal disease in diabetic retinopathy play a role (Fig. 1). One is based in a progressive retinal tissue degenerative process identified by progressive thinning of the GCL + IPL, with or without early damage to the photoreceptor layers [25]. In a five-year follow-up study, including only eyes in the early stages of DR, we identified indeed the presence of neurodegenerative changes (thinning of the GCL + IPL), in a large proportion of patients. [39]. In a large multicentre study, the EUROCONDOR, measurements related to neurodysfunction and/or neurodegeneration showed that neurodysfunction/neurodegeneration was present in 68% patients with ETDRS level 20–35 [40]. These numbers follow closely the distribution observed in our five-year follow-up study. In approximately another 25% of the patients breakdown of the BRB dominated the clinical picture whereas in another 25%, capillary closure demonstrated by decreased values of skeletonized vessel density (SVD) identified by OCTA was predominant leading to a situation of capillary non-perfusion and ischaemia.

Therefore, the presence of different major disease pathways appears to characterize the progression of diabetic retinal disease: neurodegeneration, alteration of the BRB and vessel hypoperfusion and closure (Fig. 1). These findings are supported by a series of prospective and longitudinal follow-up studies, showing that the progression of retinal disease in type 2 diabetes is associated with the predominance of the disease pathways previously described [37, 41]. These different progression phenotypes, described as A, B and C, appear to be associated with different risks for progression and development of vision-threatening complications [24].

This phenotype characterization was initially identified based on microaneurysm turnover (MAT) and central retinal thickness (CRT) measured by CFT and OCT, respectively [24]. In a five-year follow-up study, using this phenotype characterization, eyes with phenotype A and B representing approximately 75% of the mild NPDR population studied did not develop any vision-threatening complication, such as CSMO or PDR. On the other hand, PDR developed only in eyes with phenotype C [24].

**Fig. 2:**
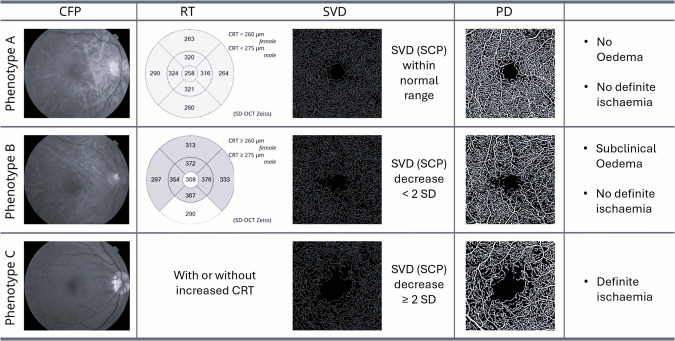
Characterization of phenotypes A, B and C of diabetic retinopathy progression.

Following on these studies and the availability of OCTA, our group has been using decreasing SVD as a more direct and objective indicator of capillary nonperfusion and ischaemia, rather than MAT, to identify the ischaemic phenotype C [42] (Fig. 2).

Another important concept that further confirms the complexity of diabetic retinal disease is that these different phenotypes of disease progression may occur simultaneously, but to a different degree, in each patient. For example, an individual patient may show in the same eye the simultaneous presence of two different disease pathways, e.g., progressive neurodegeneration and progressive ischaemia, combining phenotypes A and C. The same may occur with phenotypes B and C or A and B. In conclusion, DR appears to progress through three major disease pathways and their relative relevance is different from one patient to another, but progression to vision-threatening complications appears to occur only in phenotype C.

## Progression of the ischaemia phenotype. Identification of the hypoperfusion and hyperperfusion stages

**Fig. 3:**
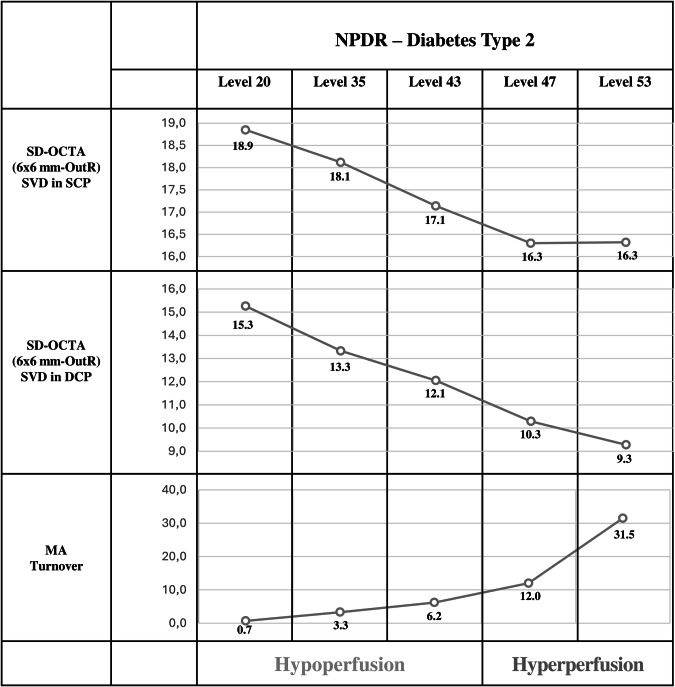
Progression of the ischaemic phenotype: capillary nonperfusion and MA turnover define hypoperfusion and hyperperfusion stages. Mean values of skeletonized vessel density (SVD) obtained with ZEISS SD-OCTA Angioplex, in superficial capillary plexus (SCP) and deep capillary plexus (DCP) in different ETDRS grade levels of NPDR mean Microaneurysm (MA) Turnover values in different ETDRS grade levels of NPDR. The hypoperfusion and hyperperfusion stages are identified.

In a series of observational follow-up studies conducted over periods of 2, 3 and 5 years, our group has therefore focused on phenotype C, characterized by predominant ischaemic changes, apparently the only phenotype that is associated with progression to vision-threatening complications (Fig. 1). This ischaemic phenotype is characterized by the presence of rapidly progressing microvascular pathology with evidence of increasing retinal vascular closure followed by increased MAT (Fig. 3) [24, 37, 39, 42–46].

Fluorescein angiography has been the gold standard imaging method to evaluate ischaemia, but it is an invasive method associated with potential complications. OCTA offers the opportunity to perform non-invasive imaging capable of providing information about capillary nonperfusion in both the superficial capillary plexus (SCP) and deep capillary plexus (DCP). Furthermore, the quality of the OCTA image is not influenced by vascular leakage, thus offering an objective way to measure the microvascular pathology [47]. Indeed, many cross-sectional and longitudinal studies have shown a good correlation between OCTA vascular metrics and DR severity [44, 48, 49].

Our studies have shown that capillary closure is an early feature of NPDR, occurs progressively and increases as retinopathy progresses (Fig. 3). It may be measured by SVD and perfusion density (PD). Our data showed that the capillary nonperfusion initially involves the SCP, progressing later to the DCP [46], confirming the results reported by Ong et al. [48].

Retinal capillary closure has been shown to be present already in the preclinical stage [23, 49] located predominantly in the central macula and increasing in subsequent ETDRS severity levels. In ETDRS level 20, the earliest stage of ophthalmoscopically visible NPDR, the capillary closure predominates in the SCP but already involving both SCP and DCP and is mainly located in the central area of the retina, the inner ring (3 × 3 mm). In ETDRS level 35 (mild NPDR), the capillary closure is identified in both retinal plexuses (SCP and DCP) and begins to extend to the outer ring of the central retina (6 × 6 mm) [45]. It is also relevant that swept-source OCTA, allowing acquisition of 15 × 15 mm images, offers an even better differentiation of the capillary closure and gives added information on the changes occurring in the midperiphery of the retina [50, 51].

Finally, in ETDRS levels 43 and 47 (moderate and moderately severe NPDR), the capillary nonperfusion is also present in both capillary plexuses and extends progressively to more peripheral areas of the retina, appearing to reach a plateau in the central retina (Fig. 3). These findings demonstrate progressive decentralization of ischaemia. To follow and quantify capillary closure or nonperfusion, OCTA measurements of the foveal avascular zone (FAZ) have also been considered. However, in our experience, FAZ metrics appear to be less reliable due to individual baseline variation.

As the retinopathy progresses, changes in capillary closure are associated with progressive increase in the number of microaneurysms and their turnover, particularly in ETDRS severity levels 43, 47 and 53 (Fig. 3). Microaneurysms are preferentially located in abnormally dilated shunt vessels, which several studies have shown to result from progressive capillary nonperfusion [52, 53]. Furthermore, the number of microaneurysms and their formation and disappearance rates appear to be good indicators of the development of IRMA, which function as dilated arteriovenous shunts. These dilated shunt vessels, first described by Cogan and Kuwabara [9], have been proposed as the main site of microaneurysm formation and appear to be particularly relevant to NPDR progression as a response to the increasing capillary nonperfusion and ischaemia (Fig. 3) [45, 54].

**Fig. 4:**
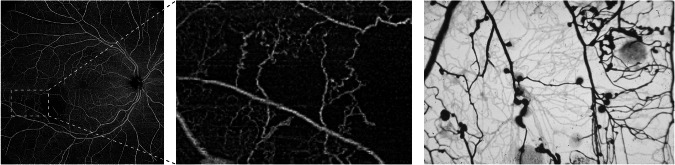
Example of retinal vascular patterns in diabetic retinopathy. On the left, a Swept-Source OCT-Angiography 15 mm × 15 mm acquisition from an ETDRS severity level 53 eye of a diabetic patient with the highlighted area magnified on the centre. On the right, a post-mortem digested retina from a different diabetic individual injected with Indian ink, showing vascular patterns characterized by capillary nonperfusion and enlarged preferential vessels (shunts) similar to those seen on the left and centre images.

These dilated preferential shunt vessels, well demonstrated in Fig. 4, appear to be responsible for the stabilization of capillary nonperfusion in the more advanced ETDRS stages and for the increasing number of microaneurysms. Ultimately, they are the precursors of IRMA which may, in turn, be the preferred sites for the development of new vessels and proliferative retinopathy [54].

Our studies have shown also the relevance of combining information on capillary closure obtained with OCTA with information on MAT as indicators of the development of shunt vessels and IRMA [50]. Capillary closure identifying the initial stage of hypoperfusion and microaneurysm counts identifying the hyperperfusion response as a result of the development of IRMAs. The goal of automated identification and discrimination of the different severity levels of NPDR appear, indeed, to be achievable by combining OCTA metrics of vessel density including information from both retinal capillary plexus (SCP and DCP) and calculation of MAT [51, 55] (Fig. 2).

As a matter of fact, in another recent study our group has been able to show that OCTA can identify significant statistical differences in OCTA metrics in eyes with advanced stages of NPDR (levels 43, 47 and 53) within a period of six months while visual function remains stable [51]. In this period of time, a clear increase in the number of microaneurysms is also identifiable in the most severe stage (level 53). It is highly relevant that OCTA and CFP are both non-invasive imaging methods, allowing risk free repeated examinations.

The capillary hypoperfusion that dominates the diabetic retinopathy severity scale (DRSS) levels 20, 35 and 43 progresses by involving the DCP until it appears to stabilize in DRSS level 53. In contrast the number of microaneurysms increases progressively through DRSS levels 47 and 53, apparently as a result of the presence of IRMA.

The observed pattern of disease progression follows the progression of DR suggested by Curtis, Gardiner and Stitt [56]. These authors have, indeed, proposed that DR progression is characterized by a hypoperfusion stage followed by a hyperperfusion response.

Our results show also that when comparing different DRSS levels in NPDR, it is possible to identify different stages of the disease by using a combination of OCTA metrics of capillary nonperfusion with microaneurysm counts. The OCTA metrics identify the progression in capillary closure and characterize the hypoperfusion stage and the increase in number of microaneurysms functioning as a surrogate for the development of dilated preferential shunts and IRMA [51].

Apparently the initial hypoperfusion stage leads to a post-ischaemic inflammatory response dominated by the establishment of dilated vessels functioning as shunts, represented by IRMA that identify the final hyperperfusion stage. It appears that it is this inflammatory response to ischaemia that leads to the development of major vision loss caused by CSMO and PDR.

The ability to monitor the balance between the initial hypoperfusion stage and the hyperperfusion response, occurring in the ischaemic phenotype of NPDR, using metrics of capillary perfusion (OCTA) and microaneurysm counting (CFP), (both non-invasive examination methods) opens new ways to predict the risk of progression to vision-threatening complications in a given eye.

The presence of predominantly peripheral lesions (PPLs) in some individuals (and their relevance to indicate disease progression) remains unclear. These peripheral lesions are better identified with widefield fluorescein angiography [57] and have been shown to correlate well with increased capillary closure in central retina identified by OCTA. However, we have shown that in the initial stages of diabetic retinal disease, some eyes show only microvascular changes in the central macular area, whereas, in others, microvascular alterations are identified only in the periphery of the retina, sparing the central retina [58]. This is clearly a topic that needs further research. Dominant peripheral retinal vascular disease in diabetes may identify a different subtype of disease progression associated with specific systemic factors, such as abnormal haematological status, considering the preferential involvement of the peripheral retinal vessels in diseases such as sick-cell disease.

Understanding which eyes of individuals with type 2 diabetes are at risk of rapid progression is clearly an unmet need. It is expected to have a major impact in the development of timely intervention to prevent vision-threatening complications.

## Vision-threatening complications: clinically significant macular oedema and proliferative diabetic retinopathy

Macular oedema can occur quite early but is most prevalent and is only associated with significant vision loss in the advanced stages of DR, when advanced ischaemic changes are already present. The Wisconsin epidemiological study demonstrated that macular oedema occurred in less than 6% of patients with mild NPDR, but this figure rose dramatically to 20-63% of patients with moderate to severe retinopathy [3]. The alteration of the BRB can occur early but is particularly vision-threatening during the hyperperfusion stage of retinopathy in association with the development of IRMA.

Proliferative retinopathy involves the formation of new blood vessels that penetrate the retinal inner limiting membrane into the vitreous. These new vessels are fragile, lead to proliferation of fibrous preretinal and vitreous haemorrhages or tractional detachment, which may result in sudden visual loss. This proliferative stage of the disease appears to be the result of the retinal vascular alterations occurring in the hyperperfusion stage of the retinopathy, which appears to be a proangiogenic response to the increasing ischaemia that dominates in the hypoperfusion stage and is the hallmark of the ischaemic phenotype C.

Both CSMO and PDR can be considered to be a result of multiple ischaemia/reperfusion episodes that occur during the progression of retinopathy [59, 60].

Our present understanding of the progress of diabetic retinal disease indicates that prevention of the major vision-threatening complications, CSMO and PDR, may be addressed by either halting the initial progressive ischaemia or by targeting the angiogenic and inflammatory response that follows a combination of both mechanisms may offer a particularly promising approach.

## Monitoring the severity of the hypoperfusion stage. OCTA metrics of capillary nonperfusion

The hypoperfusion stage of NPDR is well identified by progressive capillary nonperfusion represented by decreases in SVD and PD, which initially involve the SCP and later the DCP. There is predominant involvement of the temporal quadrant, [51]. With the progressive involvement of the DCP the capillary nonperfusion extends to the midperiphery of the retina. Perfusion deficits identified by OCTA may also be a good candidate as biomarkers of capillary nonperfusion [55].

## Monitoring the severity of the hyperperfusion stage. Microaneurysms counting as surrogate for the presence and number of IRMA

The hyperperfusion stage appears to correspond to the development of IRMA as defined by ETDRS grading standards. Quantification of IRMA, however, is difficult because of their shape and the variability of their dimensions. Our studies show that there is good correspondence between the number of microaneurysms and their turnover over a period of time and the development and increase in number of IRMA. It appears that the number of microaneurysms and their turnover can serve as surrogate for the presence and number of IRMA [51].

## Summary and future goals

Novel non-invasive imaging methods, such as OCTA, have allowed improved understanding of the progression of NPDR and the early identification of eyes at risk of progression to vision-threatening complications. The eyes that show progressive decrease in capillary perfusion, identified by decreases in SVD and PD (hypoperfusion stage), progress rapidly, leading to a hyperperfusion response, characterized by the development of IRMA and the rapid increase of the number of microaneurysms.

Our results also demonstrate that the progression of DR can be monitored using noninvasive imaging methods, such as OCTA and CFP.

The Eye Diseases Prevalence Research Group classified DR into two major outcomes, any DR, and as DR likely to result in vision loss on the absence of treatment, consisting of PDR, CSMO or both [61].

Our perspective now indicates four main outcomes, one as any DR without evidence of definite ischaemia, a second one, DR with evidence of definite ischaemia, a third one, DR with definite ischaemia and a relatively high count of microaneurysms and microaneurysm turnover and finally a fourth one identified as vision threatening DR consisting of PDR or CSMO or both.

We believe that there is, at the moment, a better understanding of the progression of NPDR, allowing the identification of the eyes at risk of rapid progression and development of vision-threatening complications (CSMO and PDR). It appears to be possible to identify if an eye presents an ischaemic phenotype and if it is still in the hypoperfusion stage or has already progressed to the hyperperfusion stage. This improved understanding of DR progression opens new opportunities to test new treatment strategies according to the stage of disease progression and predominant pathology.

## Summary

### What was known before

- Retinal ischaemia can be quantified and followed by noninvasive OCTA imaging plays a dominant role in the progression of nonproliferative diabetic retinopathy and in the development of vision-threatening complications. However, this relationship is not fully understood.

### What this study adds

- The study shows that progression of nonproliferative diabetic retinopathy to vision-threatening complications is the result of the balance between progressive capillary closure, identifying the hypoperfusion stage of the disease, and an hyperperfusion response characterised by the development of IRMAs and identified by an abnormally increased MA turnover.

- This improved understanding of diabetic retinopathy progression opens new opportunities to test novel treatments to prevent and manage complications.
